# Economic Burden of RSV-Associated Hospitalizations in Switzerland: A Nationwide Analysis (2017–2023)

**DOI:** 10.3390/healthcare14121722

**Published:** 2026-06-15

**Authors:** Maria Boesing, Daphne McCarthy-Pontier, Joerg Daniel Leuppi, Nike Julia Kräutler

**Affiliations:** 1 University Institute of Internal Medicine, Kantonsspital Baselland, 4410 Liestal, Switzerland; 2 Faculty of Medicine, University of Basel, 4056 Basel, Switzerland; 3 Moderna Switzerland GmbH, 4052 Basel, Switzerland

**Keywords:** respiratory syncytial virus (RSV), hospitalization, health economics, hospitalization costs, length of stay, older adults, aging population, vaccination Switzerland

## Abstract

**Highlights:**

**What are the main findings?**

- RSV-associated hospitalizations generate substantial annual inpatient costs in Switzerland (CHF 55–70 million).
- Older adults (≥60 years) contribute disproportionately to total costs due to higher severity and longer hospital stays.

**What are the implications of the main findings?**

- RSV represents a significant and under-recognized economic burden for healthcare systems.
- These data provide key inputs for health-economic evaluations and reimbursement decisions for RSV prevention strategies.

**Abstract:**

**Background/Objectives:** Respiratory syncytial virus (RSV) is a major cause of respiratory illness across the lifespan, yet its health-economic burden in adults remains under-recognized. Building on a previously published nationwide analysis of RSV-associated hospitalizations in Switzerland (2017–2023), this study aimed to estimate age-specific direct inpatient hospitalization costs and assess their implications for healthcare systems. **Methods:** We conducted a nationwide health-economic analysis using Swiss Federal Statistical Office (FSO) hospitalization data (2017–2023) combined with SwissDRG-based cost statistics (2024). Age-specific costs per hospitalization were applied to RSV-associated hospitalization counts. To account for disease severity, additional estimates were derived by applying RSV-specific length-of-stay (LOS) ratios between RSV-associated and all-cause hospitalizations, reflecting the longer duration of RSV-associated admissions. **Results:** Total RSV-associated hospitalization costs were estimated at CHF 55.1–76.0 million annually. Children aged 0–9 years accounted for the highest number of hospitalizations and the largest share of total costs (CHF 27.8–34.3 million). Despite fewer hospitalizations, adults aged ≥60 years generated comparable total costs (CHF 23.6–36.7 million), driven by substantially higher costs per case. Costs increased markedly with age, reflecting longer hospital stays and higher clinical severity. Additional analyses demonstrated a substantial increase in costs in the post-pandemic period, particularly in older adults, suggesting improved detection of RSV-associated hospitalizations. **Conclusions:** RSV-associated hospitalizations impose a substantial economic burden on the Swiss healthcare system. The disproportionate contribution of older adults highlights the importance of targeted prevention strategies and provides a foundation for future health-economic evaluations and policy decision-making.

## 1. Introduction

Respiratory syncytial virus (RSV) is a leading cause of acute respiratory infection across the life course and is well established as a major driver of hospitalization in infants and young children [1,2]. RSV in adults—particularly in older adults and those with multimorbidity—has historically been under-recognized, in part due to lower testing rates, lower viral loads, and frequent coding as a secondary diagnosis [3,4]. In our recent nationwide Swiss analysis using Federal Statistical Office (FSO) inpatient data from 2017 to 2023, we demonstrated a clear bimodal distribution of RSV-associated hospitalizations [5]. The highest incidence was observed in children aged 0–9 years, largely driven by infants, and a second peak emerged from the age of 50 years onwards, accompanied by an age-dependent increase in length of stay and severe outcomes, including mechanical ventilation, intensive care unit (ICU) admission, and in-hospital mortality. In adults, RSV was predominantly recorded as a secondary diagnosis in the context of chronic cardiopulmonary and metabolic comorbidities as well as frailty-associated conditions.

International health-economic analyses have shown that RSV-associated hospitalizations—particularly in older adults—are resource-intensive and associated with high direct medical costs. In the United States, hospitalization costs for RSV in adults aged ≥60 years have been reported to be comparable to or higher than those observed for influenza, reflecting longer hospital stays and higher rates of intensive care admission [6]. In Europe and Canada, analyses have estimated considerable annual healthcare and societal costs attributable to RSV in adults [3,7].

In 2024, Stucki et al. provided the first Swiss estimates of total hospitalization costs for RSV using administrative inpatient data from 2003 to 2021 [8]. They reported annual direct medical costs of approximately CHF 17–32 million for hospitalizations with RSV coded as the main diagnosis, with mean costs per hospitalization of around CHF 8700 across all age groups and substantially higher costs associated with ICU admission. While this work represents an important contribution, it did not focus on age-specific cost patterns and did not specifically address hospitalizations with RSV recorded as a secondary diagnosis, which constitute a substantial proportion of adult RSV hospitalizations.

Switzerland lacks a contemporary, age-stratified estimate of direct hospitalization costs for RSV. This gap is particularly relevant given the high burden of severe outcomes observed in older adults [9,10] and the recent availability of RSV preventive interventions [11,12,13].

The objective of the present study was to estimate age-stratified direct inpatient hospitalization costs associated with RSV in Switzerland by combining nationwide hospitalization data with age-specific cost estimates.

## 2. Materials and Methods

### 2.1. Study Design and Data Sources

This study represents a health-economic extension of a previously published nationwide analysis of RSV-associated hospitalizations in Switzerland covering the period from 2017 to 2023. The underlying hospitalization dataset and epidemiological methods have been described in detail elsewhere [5]. In brief, hospitalization data were obtained from the Swiss Federal Statistical Office (FSO) and included all inpatient admissions between 2017 and 2023 with the following RSV-related ICD-10-GM codes (primary or secondary diagnosis): B97.4 (RSV as the cause of diseases classified elsewhere), J12.1 (pneumonia due to RSV), J20.5 (acute bronchitis due to RSV), and J21.0 (acute bronchiolitis due to RSV). Age groups were primarily defined in 10-year increments; however, the oldest age category (≥80 years) was treated as an open-ended group. In addition, adults aged ≥60 years were evaluated as a combined group to reflect clinically relevant risk stratification and public health targeting of RSV prevention.

### 2.2. Hospitalization Counts and Clinical Parameters

For each age group and calendar year, absolute numbers of RSV-associated hospitalizations were included (primary or secondary diagnosis) to ensure capture of cases in adults and older populations, in whom RSV is frequently recorded as a secondary diagnosis. Clinical outcome measures (length of stay, intensive care unit admission, mechanical ventilation, and in-hospital mortality) were not recalculated in the present analysis but are referenced to contextualize cost patterns.

In addition to analyses by 10-year age groups, adults aged ≥60 years were evaluated as a combined group. This threshold reflects the age at which RSV-associated hospitalization rates, disease severity, and in-hospital mortality increase substantially and aligns with current regulatory approvals and public health recommendations for RSV prevention in Switzerland. Adults aged 20–29 years were used as the reference group for relative comparisons across adult age strata, as this group exhibited consistently low hospitalization rates and stable cost estimates.

### 2.3. Cost Data and Costing Approach

Age-specific direct hospitalization costs were estimated using publicly available inpatient cost data published by the Swiss Federal Office of Public Health (FOPH) for the year 2024 [14]. These data originate from SwissDRG-based hospital reimbursement statistics and report aggregated mean inpatient costs per case across all-cause hospitalizations, stratified by age group. Comparable data in this format are not publicly available for earlier years. Given the well-documented upward trend in hospital costs in Switzerland over time [15], the use of the most recent cost data (2024) provides a pragmatic basis for estimating the contemporary and forward-looking economic burden of RSV-associated hospitalizations.

These cost estimates were used as a standardized proxy for hospitalization costs across age groups in the subsequent analysis.

Baseline RSV-associated hospitalization costs were estimated by multiplying age-specific SwissDRG-based mean inpatient costs per hospitalization case (CHF) with the corresponding annual number of RSV-associated hospitalizations. To capture increased resource utilization associated with more severe RSV-associated admissions, a secondary, LOS-based cost estimate was derived. Age-specific RSV length-of-stay (LOS) ratios were calculated as the ratio of mean LOS for RSV-associated hospitalizations (2017–2023) to mean LOS for all-cause hospitalizations (2024). These RSV-specific LOS ratios were applied to baseline age-specific cost-per-case estimates to derive LOS-based per-case costs, which were subsequently multiplied by age-specific RSV hospitalization counts. This approach assumes that prolonged length of stay reflects increased resource utilization, consistent with previously observed higher rates of intensive care unit admission and mechanical ventilation in RSV-associated hospitalizations [5], while detailed unit cost data for these components are not separately available within national SwissDRG statistics.

SwissDRG- and LOS-based estimates were used to define a range of RSV-associated hospitalization costs. The analysis was conducted from a healthcare system perspective, focusing on direct inpatient hospitalization costs only.

### 2.4. Statistical Analysis

All analyses were descriptive in nature. Costs are presented as absolute annual totals and as mean annual costs stratified by age group. No formal statistical comparison or inferential modeling was performed, as the analysis is based on aggregated administrative data without access to patient-level variables and was designed to quantify and contextualize the magnitude of age-stratified hospitalization costs rather than to infer causality. All calculations and figures were generated using Microsoft Excel 2016.

### 2.5. Sensitivity and Scenario Analyses

To assess the robustness of cost estimates and the potential impact of temporal and diagnostic factors, additional analyses were conducted. First, a non-pandemic sensitivity analysis was performed, excluding the years 2020 and 2021, to account for reduced RSV circulation during the COVID-19 pandemic. Second, a comparison between pre-pandemic (2017–2019) and post-pandemic (2022–2023) periods was conducted to evaluate the impact of changes in testing practices and clinical awareness on RSV-associated hospitalization costs.

### 2.6. Ethical Considerations

This study was conducted in accordance with the ethical standards of the Declaration of Helsinki. Ethical approval was not required, as only aggregated and fully anonymized data were analyzed. The underlying data were collected as part of routine healthcare reporting.

## 3. Results

### 3.1. RSV-Associated Hospitalizations by Age Group

As previously reported, RSV-associated hospitalizations in Switzerland showed a pronounced age-dependent distribution, with a bimodal pattern characterized by a peak in young children and a second increase from mid-adulthood onward (Supplementary Figure S1 and Bally-von Passavant et al. [5]). Hospitalization numbers were highest in children aged 0–9 years, lowest in adolescents and young adults, and increased progressively from the age of 50 years, reaching a second peak in adults aged ≥80 years. When aggregated, adults aged ≥60 years accounted for a substantial proportion of hospitalizations despite lower absolute numbers compared with children. Marked interannual variability was observed, with a decline during the COVID-19 pandemic (2020–2021) followed by a rebound in 2022–2023.

### 3.2. Age-Specific All-Cause Hospitalizations and SwissDRG-Based Costs per Case

The mean SwissDRG-based inpatient costs per case (all-cause hospitalization) showed a progressive increase with age (Supplementary Figure S2). Costs per case were lowest in children aged 0–9 years (CHF 8116) and highest in older adults, increasing from CHF 12,175 in adults aged 20–29 years to CHF 17,589 in those aged 70–79 years and remaining similarly high in adults aged ≥80 years (CHF 16,509). Adolescents aged 10–19 years exhibited comparatively high costs per case (CHF 15,743), which likely reflects low hospitalization volumes combined with a selection of more complex or severe cases requiring inpatient care, thereby disproportionately influencing mean cost estimates in this age group, consistent with the underlying hospitalization patterns observed in our previous nationwide analysis [5].

In contrast, all-cause hospitalization volumes followed a different pattern, namely high numbers observed in young children and a marked increase again in older adults, while adolescents and young adults had consistently low hospitalization numbers. When considered alongside their lower hospitalization volumes, the overall health-economic contribution of adolescents remained limited despite higher costs per hospitalization case (Supplementary Figure S2).

### 3.3. Age-Specific RSV-Associated Hospitalization Costs per Case

**Figure 1:**
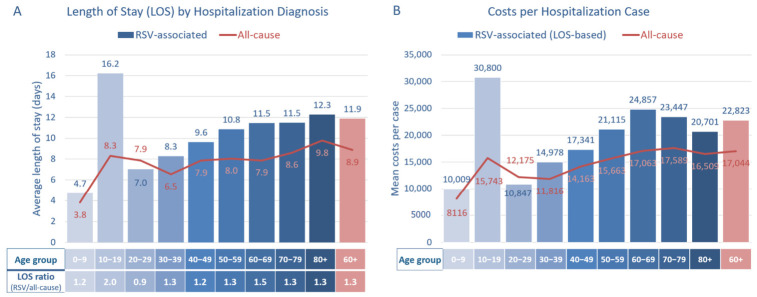
Age-specific length of stay and inpatient cost per case in Switzerland, SwissDRG-based and RSV-associated values, stratified by 10-year age groups: (**A**) age-specific length of hospital stay (RSV-associated and all-cause); (**B**) mean inpatient cost per case. SwissDRG-based values represent observed mean inpatient costs per case derived from all-cause hospitalizations (2024), whereas RSV-associated values represent LOS-based cost estimates for RSV-coded hospitalizations (2017–2023). Age-specific LOS ratios, defined as the ratio of RSV-associated to all-cause length of stay, were applied to adjust per-case cost estimates.

Across age groups, both LOS and per-case costs varied substantially (Figure 1A). For all-cause hospitalizations, mean LOS was less than 4 days in children aged 0–9 years, 7.9 days in adults aged 60–69 years, and 9.8 days in adults aged ≥80 years (Figure 1). LOS in RSV-associated hospitalizations was 0.9 to 3.6 days longer than in all-cause hospitalizations in most age groups, with 4.7 days in children aged 0–9 years, 11.5 days in adults aged 60–69 years, and 12.3 days in adults aged ≥80 years. In adolescents aged 10–19 years, comparatively higher LOS and per-case costs were observed. As noted in Section 3.2, this pattern likely reflects low hospitalization volumes and a concentration of more complex cases, thereby influencing mean estimates in this age group, consistent with the underlying hospitalization patterns observed in our previous nationwide analysis [5].

In the absence of RSV-specific cost data and detailed unit cost information for components such as intensive care or mechanical ventilation within SwissDRG statistics, age-dependent differences in LOS between RSV-associated and all-cause hospitalizations were used to derive LOS-based estimates of RSV-associated costs per case (Figure 1B). This approach approximates the additional resource utilization associated with more severe and longer RSV-associated hospitalizations.

RSV-specific LOS ratios led to higher estimated costs per hospitalization, particularly from the age of 50 years onward. After this adjustment, the estimated mean per-case costs increased from approximately CHF 8100 to 10,000 in children aged 0–9 years and from approximately CHF 17,000 to 22,800 in adults aged ≥60 years.

### 3.4. Annual RSV-Associated Hospitalization Costs

**Figure 2:**
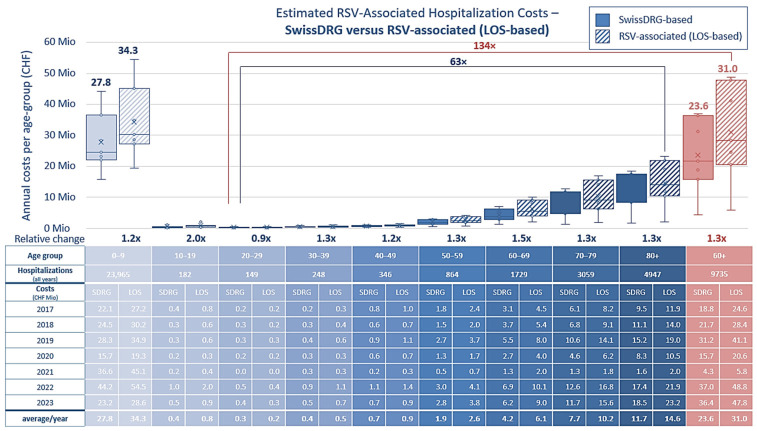
Estimated annual RSV-associated hospitalization costs by age group in Switzerland (2017–2023): SwissDRG-based versus RSV LOS-based estimates. Total annual direct inpatient costs were calculated by multiplying age-specific RSV-associated hospitalization counts with mean inpatient costs per case derived from SwissDRG-based reimbursement statistics (SDRG). RSV LOS-based estimates were derived by applying age-specific ratios of RSV-associated versus all-cause length of stay to baseline cost estimates to account for increased resource utilization associated with more severe disease. Boxplots represent the distribution of annual costs across the study period, with medians, interquartile ranges, and means (×). Values above the plots indicate mean annual costs per age group (CHF, millions). Relative changes (×) between SDRG-based and RSV LOS-based estimates are shown for each age group. Adults aged ≥60 years are additionally presented as an aggregated group.

Using age-specific RSV-associated hospitalization counts and corresponding mean SwissDRG-based inpatient costs per hospitalization case, annual total RSV-associated hospitalization costs were estimated for the period 2017–2023 (Figure 2). Across all age groups, SwissDRG-based RSV-associated inpatient costs amounted to approximately CHF 55.1 million per year. Children aged 0–9 years generated the largest share of costs, with a mean of CHF 27.8 million per year, reflecting high hospitalization volumes despite comparatively lower costs per case.

Across adult age groups, total annual costs increased steeply with age, rising from CHF 0.3 million in adults aged 20–29 years to CHF 11.7 million in those aged ≥80 years. When aggregated, adults aged ≥60 years accounted for approximately CHF 23.6 million per year, indicating a disproportionate contribution to total inpatient costs despite lower hospitalization numbers compared with children.

Incorporating prolonged hospitalization duration through LOS-based estimates in RSV-associated hospitalizations resulted in consistently higher cost estimates across age groups. In adults aged ≥80 years, costs increased from CHF 11.7 million to approximately CHF 14.6 million per year, and when aggregated, costs in adults aged ≥60 years increased from CHF 23.6 million to approximately CHF 31.0 million per year. Overall, total annual costs increased from approximately CHF 55.1 million per year to an upper-range estimate of approximately CHF 70.2 million (Figure 2).

### 3.5. Sensitivity and Scenario Analyses

To account for the potential impact of reduced RSV circulation during the COVID-19 pandemic, we conducted a sensitivity analysis excluding the years 2020–2021 (non-pandemic analysis). Excluding these resulted in higher estimated annual RSV-associated hospitalization costs. Mean annual costs increased from approximately CHF 55.1 million to CHF 61.9 million, corresponding to an increase of around 20%, with minimal impact observed in pediatric age groups but a more pronounced effect in adults (Supplementary Figure S3).

**Figure 3:**
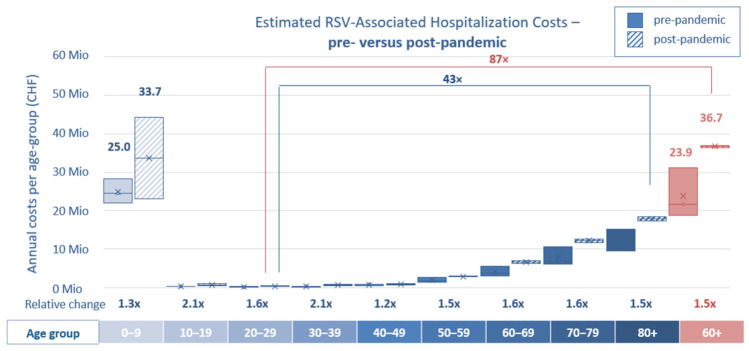
Estimated annual RSV-associated hospitalization costs by age group in Switzerland (2017–2023): pre- versus post-pandemic comparison. Total annual direct inpatient costs were calculated by multiplying age-specific RSV-associated hospitalization counts with mean inpatient costs per case derived from SwissDRG-based reimbursement statistics. Pre-pandemic estimates include the years 2017–2019, and post-pandemic estimates include the years 2022–2023. Boxplots represent the distribution of annual costs across the study period, with medians, interquartile ranges, and means (×). Values above the plots indicate mean annual costs per age group (CHF, millions). Relative changes (×) between pre- and post-pandemic estimates are shown for each age group. Adults aged ≥60 years are additionally presented as an aggregated group.

To assess whether changes in testing practices and clinical awareness may have influenced the observed burden of RSV, we compared pre-pandemic (2017–2019) and post-pandemic (2022–2023) periods (Figure 3). A marked increase in RSV-associated hospitalization costs was observed in the post-pandemic period. Total annual costs increased from approximately CHF 52.5 million to CHF 76.0 million, with relative changes ranging from approximately 1.2-fold to over 2-fold depending on age group. The largest increases were observed in older adults, indicating a substantial shift in the detected disease burden.

## 4. Discussion

We estimated age-stratified direct inpatient hospitalization costs associated with RSV in Switzerland by linking previously published hospitalization data with SwissDRG-based cost estimates and accounting for age-dependent differences in hospitalization duration. Our findings demonstrate that RSV imposes a substantial health-economic burden across the lifespan and that older adults contribute disproportionately to inpatient costs despite lower hospitalization volumes compared with young children.

Previous studies have shown that RSV-associated hospitalizations follow a pronounced bimodal age distribution, with the highest numbers observed in young children and a second peak in older adults [5,8]. Extending these observations, the present cost analysis shows that while pediatric RSV-associated hospitalizations are primarily volume-driven, hospitalizations in older adults are cost-intensive on a per-case basis. As a result, adults aged ≥60 years generated total inpatient costs comparable in magnitude to those observed in young children, despite substantially fewer hospitalizations.

A key driver of this pattern is the longer hospitalization duration observed in RSV-associated admissions among older adults. This increased disease severity in older adults is consistent with biological mechanisms such as immunosenescence and chronic low-grade inflammation associated with aging, which contribute to impaired immune responses and prolonged recovery, further underscoring the relevance of targeted preventive strategies in this population [16]. Compared with all-cause hospitalizations, RSV-associated hospitalizations were characterized by consistently prolonged LOS, translating into higher per-case costs and amplifying total inpatient expenditures. This aligns with the higher clinical severity observed in older adults, including increased rates of intensive care unit admission and mechanical ventilation in RSV-associated hospitalizations, as previously reported in the underlying nationwide analysis and in comparative studies with other respiratory viruses [5,6,17]. As detailed unit cost data for these high-intensity care components are not separately available within SwissDRG statistics, their economic impact is reflected indirectly through longer hospital stays and case-based reimbursement. Accounting for these differences increased estimated RSV-associated hospitalization costs from SwissDRG-based baseline estimates to LOS-based upper-range estimates, highlighting the importance of disease severity and resource utilization in older populations.

Importantly, the costs reported here are most likely underestimated for several reasons. Firstly, they reflect only the initial inpatient hospitalization and do not capture downstream healthcare utilization. This includes post-acute rehabilitation, outpatient follow-up, long-term care, or indirect costs related to functional decline, caregiver burden, or loss of independence—factors that are particularly relevant in older adults. Evidence from longitudinal analyses suggests that healthcare costs associated with RSV extend well beyond the acute phase, with substantial additional expenditures occurring in the months following hospitalization [18,19]. Secondly, high-cost components such as intensive care and mechanical ventilation are not separately captured in SwissDRG data. Given ICU admission rates above 15% and mechanical ventilation rates around 10% among RSV-related hospitalizations in patients aged 50 to 80 years, these represent an important cost driver in older adults that is not reflected in the estimates reported here [5].

This analysis reflects a healthcare system perspective focusing on direct inpatient hospitalization costs and does not capture RSV-specific cost components or broader societal costs. Costs were estimated based on all-cause SwissDRG reimbursement data and applied to RSV-associated hospitalizations, rather than reflecting RSV-attributable costs at the patient level. In addition, while the inclusion of both primary and secondary diagnoses improves case capture, it may also introduce heterogeneity in the attribution of costs to RSV, particularly in multimorbid patients where RSV may act as a contributing rather than primary cause of hospitalization. Finally, the use of aggregated data precludes adjustment for patient-level factors such as comorbidities or disease severity beyond LOS-based approximations.

In addition, RSV-associated hospitalizations in adults are likely underascertained due to limited diagnostic testing, lower viral loads, and frequent coding as a secondary diagnosis, leading to incomplete capture in administrative data [4,20,21,22]. While RT-PCR testing of nasopharyngeal or nasal swabs is highly specific, its sensitivity may be limited in adult populations, particularly when testing is performed later in the disease course or restricted to upper respiratory samples. A systematic review and meta-analysis reported that RSV detection increases by approximately 1.5-fold when additional diagnostic approaches, such as serology or sputum PCR, are used alongside standard nasopharyngeal RT-PCR testing [23].

The additional analyses performed in this study provide important insights into the drivers of potential underestimation of RSV-associated hospitalization costs. Excluding pandemic years resulted in a moderate increase in estimated costs, reflecting reduced RSV circulation during 2020–2021 due to public health measures. In contrast, the comparison of pre- and post-pandemic periods revealed substantially larger increases in RSV-associated hospitalization costs, particularly in older adults. This pattern is consistent with changes in testing practices and increased clinical awareness of respiratory viral infections following the COVID-19 pandemic, including broader use of multiplex PCR panels and more systematic testing of respiratory pathogens in adults, which likely improved detection of RSV infections that were previously under-recognized.

Notably, the magnitude of increase observed in the post-pandemic period exceeded the effect of LOS-based cost adjustments, suggesting that under-recognition and under-testing may contribute more strongly to underestimation of RSV burden than differences in disease severity alone.

While the inclusion of both primary and secondary diagnoses in our analysis likely improves case capture compared with studies relying exclusively on primary diagnoses, a proportion of RSV-associated hospitalizations is likely still missed due to false-negative testing and incomplete diagnostic work-up. Together, these findings suggest that the true economic burden of RSV-associated hospitalizations is likely higher than estimated here and highlight the importance of improved diagnostic strategies for accurately capturing RSV burden in adult populations.

From a public health and policy perspective, these findings are highly relevant in the context of recently introduced RSV preventive interventions. In Switzerland, prevention strategies differ by age group. For infants, protection through maternal vaccination and long-acting monoclonal antibodies has been recommended and implemented, with reimbursement in place within the mandatory health insurance framework [11,24,25,26]. In contrast, for older adults and individuals with chronic conditions, vaccination is recommended by the Swiss NITAG (EKIF) and supported by clinical societies [27,28,29], and several RSV vaccines (Abrysvo, mRESVIA, and Arexvy) have been approved by Swissmedic [11,12,13]. However, despite these recommendations and regulatory approvals, reimbursement for RSV vaccination in adults is currently not broadly implemented under the Swiss health insurance system (KLV), following assessment by the Federal Commission for Benefits and Principles (ELGK) [30]. This discrepancy between clinical recommendations and reimbursement may represent a barrier to widespread implementation. The age-stratified cost estimates presented here provide essential baseline inputs for future health-economic and outcomes research (HEOR), including cost-effectiveness and budget impact analyses of RSV prevention strategies in older populations.

## 5. Conclusions

RSV-associated hospitalizations in Switzerland generate substantial direct inpatient costs across all age groups. Although young children account for the highest number of hospitalizations, older adults aged ≥60 years incur total hospitalization costs of similar magnitude due to markedly higher costs per case. Combined with the previously demonstrated higher disease severity and mortality in older adults, these findings identify RSV as a major and under-recognized health-economic burden in aging populations. These results support the need for targeted RSV prevention strategies and provide an important basis for future health-economic evaluations and policy decisions in Switzerland.

## Data Availability

The data presented in this study are available in the Federal Statistical Office (FSO) at https://stats.swiss/vis?lc=de&df[ds]=disseminate&df[id]=DF_GVS_INPATIENT_ENCOUNTER&df[ag]=CH1.GVS&df[vs]=1.0.0&dq=_T..._T..A&lom=LASTNPERIODS&lo=1&to[TIME_PERIOD]=false&vw=tb (accessed on 16 April 2026).

## Supplementary Materials

The following supporting information can be downloaded at: https://www.mdpi.com/article/10.3390/healthcare14121722/s1, Figure S1: Mean annual RSV-associated hospitalizations by age group in Switzerland, 2017–2023; Figure S2: Age-specific all-cause hospitalization volumes and costs per case in Switzerland, 2024 (SwissDRG); Figure S3: Estimated annual RSV-associated hospitalization costs by age group in Switzerland (2017–2023): all-years versus non-pandemic estimates.

## Abbreviations

The following abbreviations are used in this manuscript:

CHF	Swiss Francs
COVID-19	Coronavirus disease 2019
DRG	Diagnosis-Related Group
EKIF	Eidgenössische Kommission für Impffragen (Federal Commission for Vaccination Recommendation)
FOPH	Swiss Federal Office of Public Health
FSO	Swiss Federal Statistical Office
ICD-10-GM	International Statistical Classification of Diseases and Related Health Problems, 10th Revision, German Modification
ICU	Intensive care unit
LOS	Length of stay
NITAG	National immunization technical advisory group
RSV	Respiratory syncytial virus
SwissDRG	Swiss Diagnosis-Related Groups
WHO	World Health Organization
