# Supplementary material for: Economic Burden of RSV-Associated Hospitalizations in Switzerland: A Nationwide Analysis (2017–2023)
Source: Healthcare (Basel). 2026 Jun 15;14(12):1722. doi: 10.3390/healthcare14121722 (PMC13300369; doi:10.3390/healthcare14121722)
Supplement: Supplementary file 1 [file healthcare-14-01722-s001.zip › healthcare-4291574-supplementary.pdf]

Supplementary

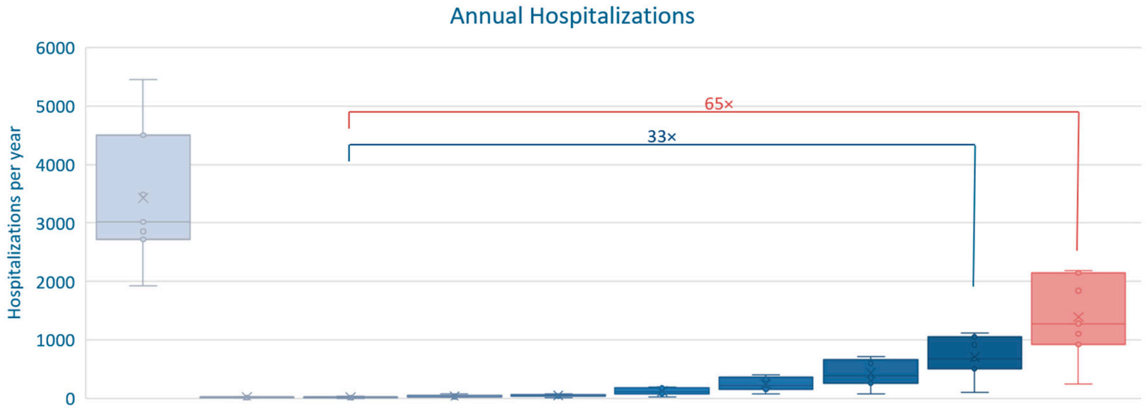

Age group	0–9	10–19	20–29	30–39	40–49	50–59	60–69	70–79	80+	60+
Hospitalizations (all years)	23,965	182	149	248	346	864	1729	3059	4947	9735
2017	2720	26	18	19	58	113	181	349	577	1107
2018	3021	19	21	28	43	93	219	386	674	1279
2019	3490	21	25	38	61	174	323	600	918	1841
2020	1930	10	14	22	40	82	159	263	505	927
2021	4505	12	2	23	16	31	79	76	98	253
2022	5445	64	39	72	78	192	405	718	1056	2179
2023	2854	30	30	46	50	179	363	667	1119	2149
average/year	3424	26	21	35	49	123	247	437	707	1391

Figure S1. Mean annual RSV-associated hospitalizations by age group in Switzerland, 2017–2023. Annual absolute numbers of RSV-associated hospitalizations stratified by age group in 10-year increments (0–9, 10–19, 20–29, 30–39, 40–49, 50–59, 60–69, 70–79, ≥80 years), with adults aged ≥60 years shown as an aggregated group for comparison. Boxplots summarize the distribution of annual hospitalization counts across the study period, showing medians, interquartile ranges, minimum and maximum values, and means (×). The table below the figure presents absolute hospitalization counts per year and age group, as well as mean annual values.

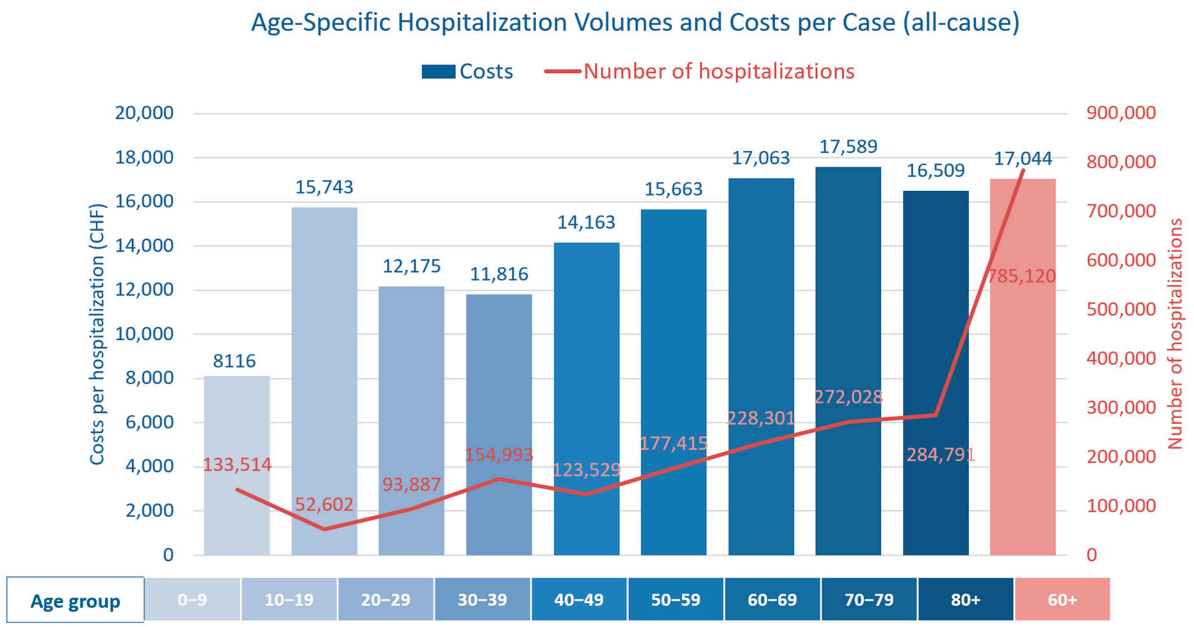

Figure S2. Age-specific all-cause hospitalization volumes and costs per case in Switzerland, 2024 (SwissDRG). Bars represent age-specific mean inpatient costs per case, and the red line indicates the number of all-cause inpatient hospitalizations by age group. These data provide background context for subsequent estimation of RSV-associated hospitalization costs.

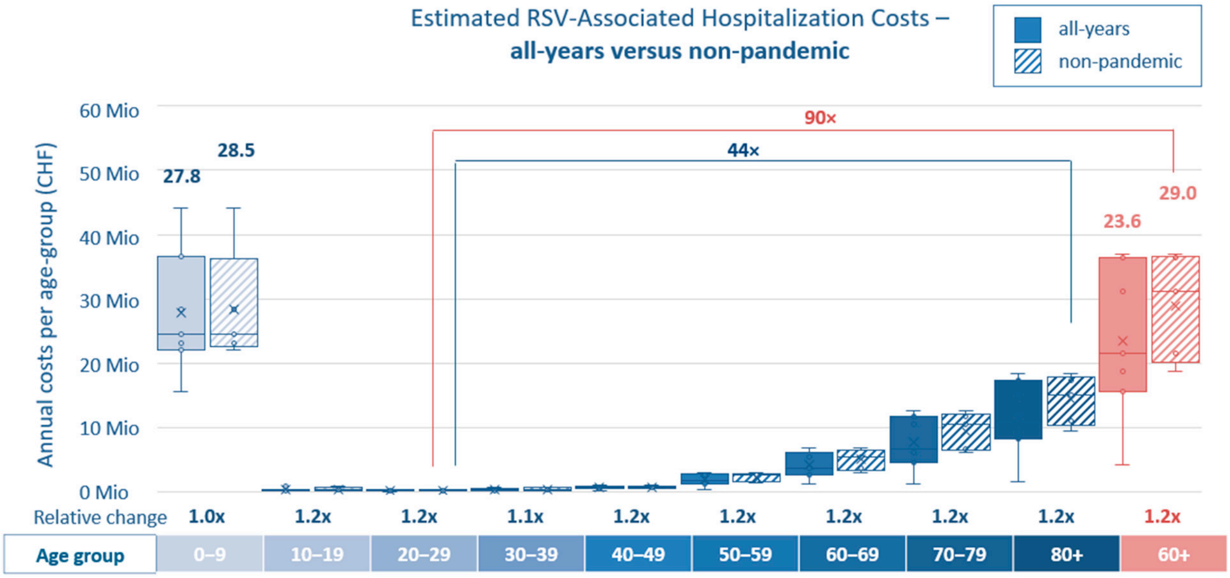

Figure S3. Estimated annual RSV-associated hospitalization costs by age group in Switzerland (2017–2023): all-years versus non-pandemic estimates. Total annual direct inpatient costs were calculated by multiplying age-specific RSV-associated hospitalization counts with mean inpatient costs per case derived from SwissDRG-based reimbursement statistics. The non-pandemic estimates exclude the years 2020–2021 to account for reduced RSV circulation during the COVID-19 pandemic. Boxplots represent the distribution of annual costs across the study period, showing medians, interquartile ranges, minimum and maximum values, and means (×). Values above the plots indicate mean annual costs per age group (CHF, millions). Relative changes (×) between all-years and non-pandemic estimates are shown for each age group. Adults aged ≥60 years are additionally presented as an aggregated group.
